# Long-Term Hypermethylation of *FcγR2B* in Leukocytes of Patients with Kawasaki Disease

**DOI:** 10.3390/jcm10112347

**Published:** 2021-05-27

**Authors:** Ling-Sai Chang, Hong-Ren Yu, Chiao-Lun Chu, Kuang-Den Chen, Ying-Hsien Huang, Mindy Ming-Huey Guo, Ken-Pen Weng, Ho-Chang Kuo

**Affiliations:** 1 Department of Pediatrics and Kawasaki Disease Center, Kaohsiung Chang Gung Memorial Hospital, Chang Gung University College of Medicine, Kaohsiung 83301, Taiwan; joycejohnsyoko@gmail.com (L.-S.C.); yuu2004taiwan@yahoo.com.tw (H.-R.Y.); o911079251@gmail.com (C.-L.C.); yhhuang123@yahoo.com.tw (Y.-H.H.); mindymhguo@yahoo.com.tw (M.M.-H.G.); 2 Institute for Translational Research in Biomedicine, Kaohsiung Chang Gung Memorial Hospital, Kaohsiung 83301, Taiwan; dennis8857@gmail.com; 3 Congenital Structural Heart Disease Center, Department of Pediatrics, Kaohsiung Veterans General Hospital, Kaohsiung 813, Taiwan; 4 School of Medicine, National Yang Ming Chiao Tung University, Taipei 711, Taiwan; 5 Department of Physical Therapy, Shu-Zen College of Medicine and Management, Kaohsiung 821, Taiwan

**Keywords:** *FcγR2B* methylation, IVIG resistance, Kawasaki disease

## Abstract

The Fc gamma receptor family contains several activating receptors and the only inhibitory receptor, *FcγR2B*. In this study, we investigated the dynamic methylation change of *FcγR2B* in different stages of Kawasaki disease (KD). We enrolled a total of 116 participants, which included patients with febrile diseases as controls and KD patients. Whole blood cells of KD patients were collected prior to intravenous immunoglobulin (IVIG) treatment (KD1), three to seven days after IVIG (KD2), three weeks after IVIG treatment (KD3), six months after IVIG (KD4), and one year after IVIG treatment (KD5). In total, 76 KD patients provided samples in every stage. Leukocytes of controls were also recruited. We performed DNA extraction and pyrosequencing. *FcγR2B* methylation levels were higher in KD3 compared to both the controls and KD1. A significantly higher methylation of *FcγR2B* was found in KD5 when compared with KD1. *FcγR2B* methylation levels in the IVIG-resistant group were lower than those in the IVIG-responsive group at KD1-3 (*p* = 0.004, 0.004, 0.005 respectively). This study is the first to report the dynamic change of *FcγR2B* methylation and to demonstrate long-term hypermethylation one year after disease onset. Hypomethylation of *FcγR2B* is associated with IVIG resistance.

## 1. Introduction

Kawasaki disease (KD) is a form of vasculitis characterized by such symptoms as conjunctivitis, strawberry tongue or erythema and cracking lips, skin rash, erythema and edema of the limbs, and cervical lymphadenopathy, in addition to a fever lasting at least five days, for the primary diagnostic criteria [1,2]. An association between Fc receptors (FcR), inflammation, and KD has already been well-recognized [3,4]. FcγRIIB encoded by *FcγR2B* is the only inhibitory FcR and is expressed on neutrophils, eosinophils, mast cells, memory B cells, plasma cells, monocytes, and macrophages [5,6]. Meanwhile, Fc gamma receptors (FCGRs) play a vital role in mediating the inflammatory suppression by intravenous immunoglobulin (IVIG) through a Th2 pathway [7,8]. Xia et al. previously identified lower FcγRIIB expression on monocytes in KD patients with coronary artery aneurysms (CAAs) and the expression increased following IVIG treatment [7]. They observed that interleukin (IL)-6 and TNF-α mRNA in monocytes had a negative correlation with FcγRIIB expression on monocytes. Genetic studies have shown that functional *FcγR2B* gene variants *FcγR2B (-120T/A)* influence IVIG response in patients with KD in Caucasians [9]. Shrestha et al. demonstrated a higher likelihood of being responders with a greater number of A allele, which generated higher receptor expression as a result of promoter activity confirmed by in vitro luciferase reporter assays before [10,11]. However, the A allele at *FcγR2B-120T/A* was completely absent in Asian groups.

In our previous study, we reported no significant difference in the mRNA expression of *FcγR2B* in leukocytes between KD patients and controls [12]. Furthermore, the expression levels of *FcγR2B* mRNA did not differ significantly in leukocytes of KD patients with regard to CAA formation or IVIG treatment response in samples collected from pre-IVIG to three weeks after recovery from KD. Previous studies showed that IVIG increased FcγRIIB expression on monocytes/macrophages by type 2 inflammatory cytokines IL-4 in mice while others showed that this was not the case in KD patients [8,13].

DNA methylation, which is among the primary epigenetic mechanisms, has been implicated in the pathogenesis of KD. Our previous study demonstrated that the methylation of *FcγR2A,* a low-affinity activating FCGR, was lower in patients with KD and in those KD patients with IVIG resistance [14]. These findings supported higher *FcγR2A* transcriptional levels in KD patients. By using methylation array, our previous study revealed the upregulation of *FcγR2B* methylation following IVIG infusion in KD [12]. However, there is no literature tracking *FcγR2B* methylation in KD patients with a long-term follow-up. Whether these epigenetic modifications have long-term persistence is unclear, but this question is important since KD patients often develop type 2 inflammation-related allergic diseases later in life [15,16]. IL-4 levels were higher in KD compared to controls. The essential role of DNA methylation is long-term transcriptional regulation according to other models. For example, sublingual immunotherapy decreased DNA methylation significantly in memory regulatory T cells after one year of treatment [17,18]. The current study aimed to investigate the long-term epigenetic changes of the *FcγR2B* gene in KD.

## 2. Methods

### 2.1. Subjects

**Figure 1:**
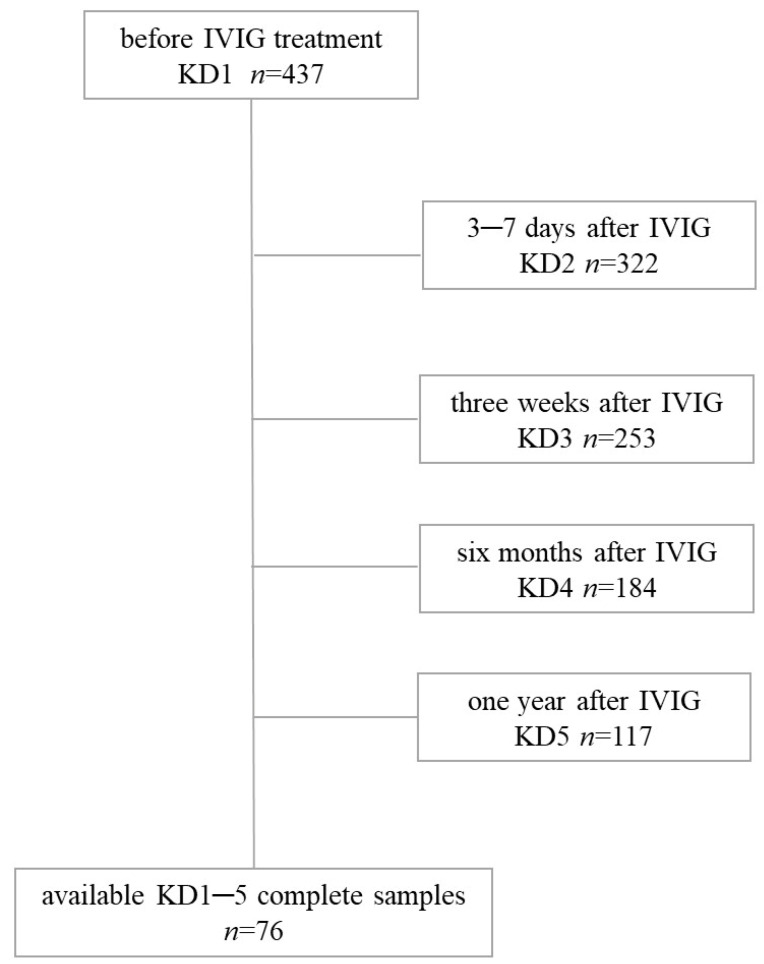
Flowchart of patients with Kawasaki disease. KD, Kawasaki disease; IVIG, intravenous immunoglobulin.

For this prospective study, we recruited 116 subjects from Kaohsiung Chang Gung Memorial Hospital, a major medical center in Taiwan. Of those, 40 subjects belonged to the control group, which consisted of patients who had fever but were not diagnosed with KD. We recruited patients with febrile diseases, including bacterial or viral infections as controls, but we excluded any patients with underlying diseases or congenital diseases, including neurological diseases, hematologic diseases, congenital heart diseases, or malignant tumors. The other 76 subjects were KD patients diagnosed and hospitalized in the ward according to the KD diagnostic criteria set forth by the American Heart Association (AHA) [2]. The diagnostic criteria of KD included any child with a fever lasting more than five days and four of the following five criteria: diffuse mucosal inflammation of the tongue and fissure lips, bilateral non-purulent conjunctivitis, angioedema over limbs, multiple rashes, and unilateral cervical lymphadenopathy [19]. According to the guidelines of the AHA, all patients with KD were treated with high-dose IVIG (2 g/kg) [20]. Inclusion criteria for the study were *FcγR2B* methylation measured at all five stages (Figure 1). Transient dilatations and incomplete KD patients were also included in the study. The exclusion criteria were *FcγR2B* methylation tests < 5 times or treatment at other hospitals. The 76 KD patients who were admitted to the hospital and received all five *FcγR2B* methylation tests were subjected to analysis in this study for kinetic changes of *FcγR2B* methylation after discharge. This study’s IVIG resistance subjects included six IVIG-resistant individuals. This study was approved by the institutional review board of Chang Gung Memorial Hospital and carried out in accordance with the Helsinki Declaration (IRB No. 104-9786A3). After providing a detailed explanation of the study, we obtained written informed consent from the parents or guardians of all participants. IVIG responsiveness (IVIG sensitivity) was defined as fever subsided within 48 h and then no fever (>38 °C) for seven days or more following initial IVIG treatment. We used two-dimensional cardiac ultrasound to determine the presence of CAA, which we defined as a coronary artery with a diameter greater than 3 mm or a diameter greater than 4 mm in patients over 5 years old or an internal diameter of a segment at least 1.5 times the size of an adjacent segment, or a z-score ≥2.5. We performed such laboratory tests as complete blood count (CBC), differential counts, and C-reactive protein (CRP) tests on the 76 children with KD.

### 2.2. Measurement of DNA Methylation Levels of FcγR2B Promoter Regions by Bisulfite Pyrosequencing

**Figure 2:**
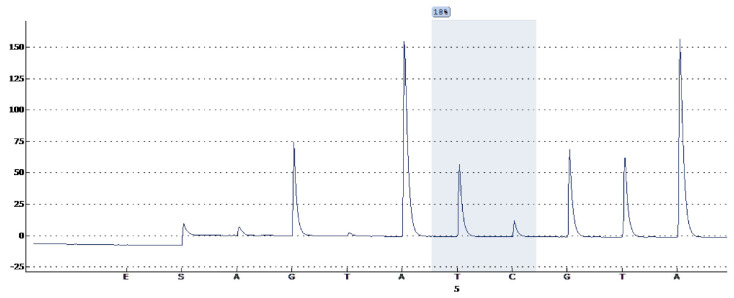
Only one methylation site was identified using pyrosequencing for target-specific sequencing at cg22436411 in the *FCGR2B* promoter region.

White blood cells were obtained from a total of 40 controls and 76 KD patients who participated in the study at KD1 to KD5. To extract DNA, we first treated the collected blood cells with a 0.5% SDS lysis buffer and then with protease K (1 mg/mL) for 4 h at 60 °C to digest the nuclear protein. DNA was isolated from blood samples using Non-Organic Solvent Reagents Purification in accordance with the manufacturer’s instructions. The DNA methylation of *FcγR2B* was measured using pyrosequencing. We further evaluated the relationship between *FcγR2B* methylation and patients’ clinical outcomes, including response to IVIG and CAA. As shown in Figure 2, one CpG methylation locus cg22436411 (Chr1, 159898477) was predicted by Pyromark Q24 software (Qiagen Inc., Valencia, CA, USA). One methylation site was identified using pyrosequencing for target-specific sequencing at cg22436411 in the *FcγR2B* promoter region. In short, 0.5 μg of genomic DNA were bisulfite modified using an EZ DNA methylation kit (Zymo Research, Irvine, CA, USA) and eluted in 20 μL of Tris buffer (10 mM) as previously described in another study [8]. Pursuant to the instructions, we used a polymerized chain reaction in 25 μL of bisulfite-converted DNA, 1×PyroMark PCR Master Mix (Qiagen Inc., Valencia, CA, USA), and 0.2 μM 25 μL of reaction mixture. The biotinylated primer of *FcγR2B* was 5’-ATGTTGAGGGTGAATAAATGG (forward) 5’-ATATTCCCCAAAAAATAAATTACCCCTAAC (reverse). After amplification, we purified and treated the product with a sequencing primer 5’-GGTAATGAGGATGATGATA, which was designed to bind adjacent to the CpG site of interest. We then applied the PyroMarkQ24 instrument and calculated the percentage of methylation using the software Qiagen 1.0.10 (Qiagen Inc., Valencia, CA, USA). 

### 2.3. Cell Culture

THP-1 cells purchased from the American Type Culture Collection (TIB-202) were maintained in a humidified atmosphere of 5% CO_2_ at 37 °C [21]. The cells were seeded at a density of 1 × 10^6^ cells in six-well plates and cultured in a fetal bovine serum/antibiotics-containing RPMI 1640 medium. The THP-1 cells were pretreated with/without 0.1 μg/mL lipopolysaccharide (from *Escherichia coli*, 055:B5, Sigma-Aldrich, Inc., St. Louis, Missouri, USA) and then either maintained as the control or had 5 mg/mL IVIG further added. THP-1 cells were cultured for 2, 4, 6, and 8 h [22]. The cells were collected for DNA extraction and pyrosequencing subject to *FcγR2B* methylation analysis after culture at the indicated times.

### 2.4. Statistical Analysis

The data in this study is expressed as mean ± standard error (Mean ± SE). We analyzed the discontinuous variables of demographic data with Chi-square and analyzed the continuous variables with Student’s *t*-test, which we further employed to calculate the *p*-values of *FcγR2B* methylation between the patients and controls. Continuous data were analyzed using the corresponding non-parametric method, the Mann-Whitney *U* test, for *FcγR2B* methylation between the IVIG response and resistance groups. Statistical significance was calculated for change of *FcγR2B* before and after IVIG using the paired *t*-test. We utilized Pearson correlation to analyze the data between *FcγR2B* methylation and laboratory tests, including CBC, differential counts, and CRP. A *p*-value less than 0.05 was considered statistically significant. All analyses were performed using SPSS software (SPSS 14.0; SPSS, Inc., Chicago, IL, USA). We repeated the in vitro experiments at least three times. The statistical analysis was completed using analysis of variance (ANOVA) followed by Fisher’s Least Significant Difference test for multiple comparisons.

## 3. Results

### 3.1. Study Subjects

This study’s participants included 40 unrelated febrile individuals (21 male; 52.50%) and 76 KD subjects (42 male; 55.26%) that met the criteria for KD diagnosis. The flow diagram for the identification of KD patients is provided in Figure 1. IVIG at 2 g/kg in a single infusion were administered to 76 (100%) patients, including 6 IVIG-resistant KD patients who had persistent or recurring fever at least 36 h after the finishing of IVIG afflux and 70 IVIG-responsive KD patients [2]. Of the 76 KD patients enrolled in this study, 18 had CAA. As shown in Table 1, the gender distribution between the two groups demonstrated no significant difference, and the age of the KD patients (1.79 ± 0.14) had no significant difference when compared with the control subjects (2.29 ± 0.26, *p* = 0.10).

### 3.2. Methylation Levels of FcγR2B Promoter Regions on Leukocytes in Different Stages of Kawasaki Disease

**Figure 3:**
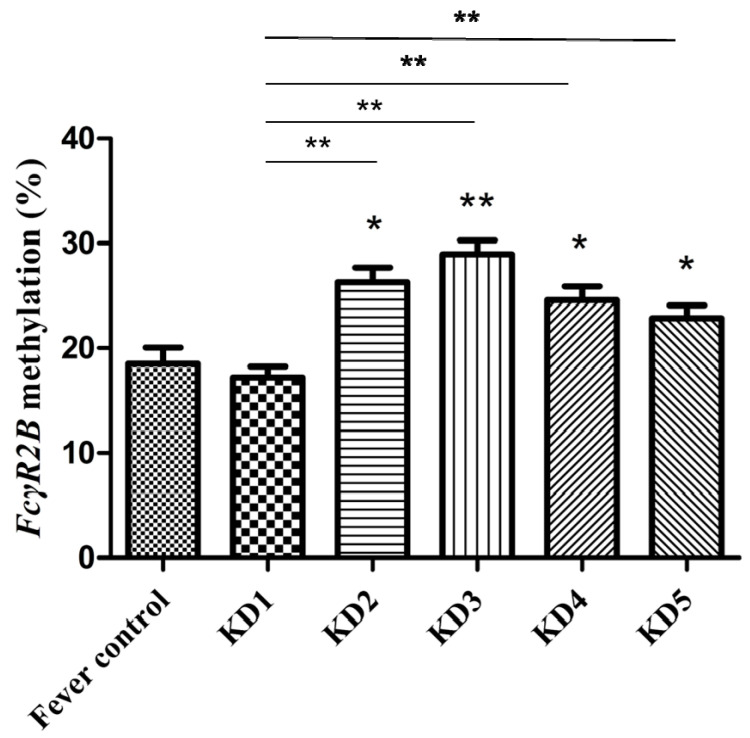
Prior to therapy, *FcγR2B* methylation did not differ significantly between Kawasaki disease (KD) and controls. After receiving intravenous immunoglobulin (IVIG) treatment, *FcγR2B* methylation increased and could last up to one year later. * denotes significance (* *p* < 0.05, ** *p* < 0.001). KD1, within 24 h prior to IVIG treatment; KD2, 3–7 days after IVIG; KD3, three weeks after IVIG, KD4, six months after IVIG; KD5, one year after IVIG.

We studied the *FcγR2B* methylation of the total white blood cells in paired blood samples from 76 KD patients (see Figure 3). We obtained peripheral blood samples from all 76 KD subjects at five different times: within 24 h prior to IVIG treatment (KD1), 3–7 days after IVIG (KD2), three weeks after IVIG (KD3), six months after IVIG (KD4), and one year after IVIG (KD5) [23]. We found no significant difference in *FcγR2B* methylation between KD patients and controls. *FcγR2B* methylation was significantly higher in samples taken three days after IVIG than in the first samples taken before IVIG (KD2: 26.29  ±  1.40 and KD1: 17.22  ±  1.02, paired *t* test, *p* < 0.001). The highest *FcγR2B* methylation was observed in KD3 samples (KD3: 28.96  ±  1.31, *p* = 0.015 when compared with KD2; *p* < 0.001 when compared with KD1). In KD5 patients, the *FcγR2B* methylation was still higher than in the children at KD1 (KD5: 22.86  ±  1.24, paired *t* test, *p* < 0.001). Many factors seemed to influence *FcγR2B* methylation, including IVIG, age, and lymphocyte counts. To rule out the possible effect of age on methylation levels, we checked the Pearson correlation between age and methylation levels, which demonstrated a negative correlation (*p* = 0.008). A generalized estimating equation was evaluated for correlations between multiple measures of the same KD patients using Bonferroni correction [24]. The methylation of *FcγR2B* increased significantly, after adjusting for lymphocyte counts and age (KD2, 3, 4, 5 vs. KD1: β = 8.43, 10.65, 8.58, 11.40; all *p* < 0.001).

Patients had similar *FcγR2B* methylation between incomplete (*n* = 15) and complete (*n* = 61) patients at KD1 (*p*  = 0.198). Of the 15 patients with steroids, steroids were administered in 10 patients after KD2. The KD3 *FcγR2B* methylation was significantly associated with KD1 *FcγR2B* methylation but not steroid usage by linear regression with KD1 *FcγR2B* methylation and steroid usage as covariates. No difference in methylation was found when clustered by years or different IVIG brands: Gamunex-C (Grifols Therapeutics, California, USA) and “TBSF” Human Immunoglobulin (Taiwan Blood Services Foundation, Taiwan) (*p* = 0.055 by the Kruskal-Wallis test; *p* = 0.336 by the Mann-Whitney *U* test) at KD2.

We discovered other CpG sites, cg22942846 and cg00831970, of *FcγR2B* from the M450 methylation array reported in our previous study [25]. There were no significant changes in methylation of the CpG sites cg22942846 and cg00831970 between non-febrile and febrile controls or non-febrile controls and KD.

### 3.3. FcγR2B Hypomethylation with IVIG Resistance in Kawasaki Disease

**Figure 4:**
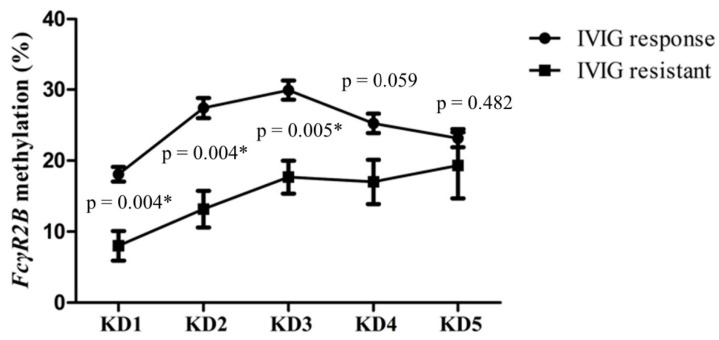
*FcγR2B* methylation was lower in the intravenous immunoglobulin (IVIG)-resistant group than in the IVIG-responsive group. KD, Kawasaki disease; KD1, within 24 h prior to IVIG treatment; KD2, 3–7 days after IVIG; KD3, three weeks after IVIG, KD4, six months after IVIG; KD5, one year after IVIG. * *p* < 0.05.

Patients with IVIG resistance (*n* = 6) had significantly lower *FcγR2B* methylation (7.33 ± 2.12 vs. 18.07 ± 1.04, *p* = 0.004) than the IVIG-responsive group (*n* = 70) (Figure 4). In IVIG non-responders, the significantly reduced *FcγR2B* methylation persisted to KD3. *FcγR2B* methylation levels in the IVIG-resistant group were lower than those in the IVIG-responsive group both 3–7 days and three weeks after IVIG (*p* = 0.004, 0.005). No significant correlation was found between *FcγR2B* methylation levels and coronary artery abnormalities in KD1, KD2, KD3, KD4, or KD5 (*p* = 0.509, 0.169, 0.219, 0.666, and 0.754, respectively). Analysis revealed that the *FcγR2B* methylation levels in the control subjects did not differ from those in KD patients with CAA (*p*  = 0.366).

### 3.4. Association between FcγR2B Methylation and Blood Parameters in Kawasaki Disease

We observed correlations between the lymphocyte proportion and acute or convalescent *FcγR2B* methylation in KD1, KD2, KD3, KD4, and KD5 (all *p* values < 0.001). We found that the proportion of segmented neutrophils was inversely correlated with *FcγR2B* methylation in KD1, KD2, KD3, KD4, and KD5 (all *p* values < 0.001), as well as total white blood cells in KD1, KD2, KD3, and KD5 (*p* = 0.023, 0.002, 0.046, 0.026). Furthermore, a reduced neutrophil-to-lymphocyte ratio was associated with *FcγR2B* methylation in KD1, KD2, KD3, KD4, and KD5 (*p* < 0.001 in KD15, respectively). Meanwhile, in patients with KD, *FcγR2B* methylation positively correlated with platelet counts (*p* = 0.047). Interestingly, this study identified a significant (*p* = 0.003) inverse association between CRP levels and *FcγR2B* methylation.

### 3.5. Hematological Changes in Kawasaki Disease

**Figure 5:**
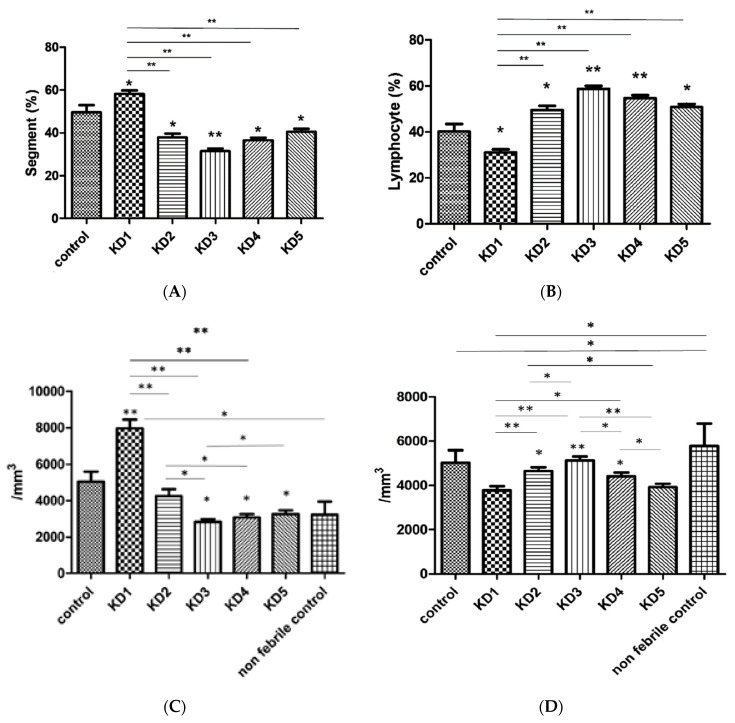
Patients treated with intravenous immunoglobulin (IVIG) developed the lowest significant decrease in percentage of segmented neutrophils and an increase in the percentage of lymphocytes by the third week. (**A**) Segmented neutrophils were statistically significantly higher in the KD group compared with controls and decreased after receiving immunoglobulin for more than one year. (**B**) The percentage of lymphocytes was significantly lower in the KD group than in the control group, increasing after receiving immunoglobulin, and the increase lasted for more than one year. Asterisks denote significance compared with controls. (**C**) Absolute neutrophil counts. (**D**) Absolute lymphocyte counts. KD1, within 24 h prior to IVIG treatment; KD2, 3–7 days after IVIG; KD3, three weeks after IVIG, KD4, six months after IVIG; KD5, one year after IVIG. * *p* < 0.05, ** *p* < 0.001.

A considerable increase of segmented neutrophils was observed in KD patients compared with controls (*p* = 0.026) (Figure 5A). In the post-IVIG stage, significant reductions were observed in segmented neutrophils (58.18 ± 1.63% in KD1, 37.81  ±  1.89% in KD2, 31.40 ± 1.29% in KD3, 36.50 ± 1.26% in KD4, 40.50 ± 1.41% in KD5) while lymphocytes were significantly lower in patients with KD compared to controls (*p* = 0.012) (Figure 5B). We observed a significant increase of lymphocytes following IVIG treatment, and lymphocytes had the most significant increase at week three (KD2 vs. KD3 *p* = 0.015). This pattern of lymphocyte changes was almost identical in *FcγR2B* methylation, both exhibiting similar increases and their highest values in KD3. After IVIG treatment, the absolute counts of neutrophils and lymphocytes in KD patients returned to the levels of the six age- and gender-matched non-febrile controls (Figure 5C,D).

### 3.6. IVIG Regulates FcγR2B Methylation in THP-1 Cells

**Figure 6:**
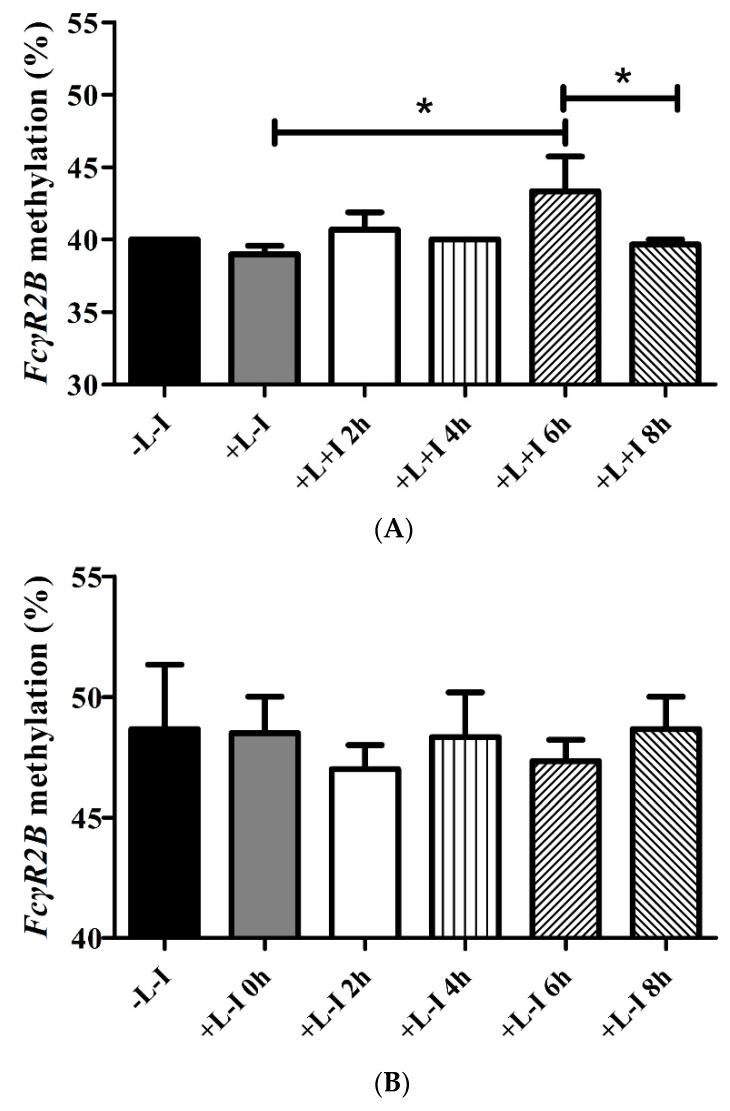
(**A**) At 6 h, upregulated *FcγR2B* methylation was detected, compared with THP-1 cells without IVIG supplement. IVIG significantly increased the *FcγR2B* methylation in the LPS-treated THP-1 cells. (**B**) No change was detectable in THP-1 cells pretreated only with lipopolysaccharide at different time points. H, hours; I, intravenous immunoglobulin; L/LPS, lipopolysaccharide (* *p* < 0.05).

Due to the stronger inhibitory effect of IVIG on the activation of monocytes than that of T cells and patients’ different expressions of *FcγR2B* on monocytes, we investigated whether IVIG could regulate *FcγR2B* methylation in THP-1 cells (a human monocytic leukemia cell line) [7,22]. Pretreating cells with IVIG and lipopolysaccharide for six hours significantly increased *FcγR2B* methylation in THP-1 cells (*p* = 0.019) (Figure 6).

## 4. Discussion

Interestingly, we found that hypermethylation at the *FcγR2B* promoter persisted for more than one year in KD. FcγRIIB is generally considered to be the only inhibitory Fc receptor that can suppress an inflammatory response. Hypomethylation of *FcγR2B* is associated with IVIG resistance, indicating that greater *FcγR2B* expression is required for an inflammatory condition in patients with IVIG resistance to overcome excessive inflammation. *FcγR2B* may play a role in downregulating inflammation. Huang et al. found that DNA methyltransferase (DNMT1) was downregulated in patients with KD, and after three days of IVIG in patients with CAA, DNMT1 was lower than patients without CAA [26]. Our previous report identified IVIG resistance was involved in a scoring system for predicting CAA [27]. Therefore, we considered that the decrease in methylation of *FcγR2A* and *B* in patients with IVIG resistance may be due to the influence of DNMT1 [14].

Although the anti-inflammatory process is usually confined to the acute phase of the IVIG response, long-term immune changes may persist following IVIG treatment. An in vitro study was designed to explore the potential role of IVIG for *FcγR2B* methylation. We found significantly higher *FcγR2B* methylation after administering IVIG and confirmed the effect of IVIG on methylation changes suspected by the analysis of Illumina HumanMethylation27 BeadChip [12,28]. Evidence supporting the existence of long-term chronic inflammation and anti-inflammation is limited, so the mechanisms responsible for the chronic immune response in KD patients and their potential consequences on patients’ health remain unknown. Our results suggest that an epigenome at specific genes that control anti-inflammation stably changes throughout the course of KD. Persistent inflammation with higher high-sensitivity CRP levels was observed in patients with KD history [29]. Part of the proposed mechanism for durable epigenetic modification may come from the therapeutic IVIG. The mechanism of sustained epigenetic modification in KD needs further research to draw a clear conclusion.

A recent study found that a neutrophil-to-lymphocyte ratio ≥ 3.2 could predict IVIG resistance [30]. An earlier study found that IVIG-resistant patients had significantly lower platelet counts than IVIG-responsive patients [31]. These results were consistent with another finding of this study that the neutrophil-to-lymphocyte ratio, lymphocytes, segmented neutrophils, and platelets were all associated with *FcγR2B* methylation. The present study suggested that *FcγR2B* methylation was lower in the leukocytes of patients with IVIG-resistant KD than in IVIG-responsive subjects. In summary, these observations support the perspective that all of these features are unique to KD patients with IVIG resistance.

Furukawa et al. observed no significant difference in lymphocytes between the matched controls and KD (4.68 ± 0.25 vs. 4.06 ± 0.20 × 10^9^/L), and lymphocyte values did not differ between KD patients in the acute stage and in the convalescent stage (4.06 ± 0.20 vs. 4.89 ± 0.19 × 10^9^/L) [32,33]. Nevertheless, the long-term anti-inflammatory effect of IVIG is still being investigated. One study found that total lymphocyte counts between both groups with IVIG-and-aspirin versus aspirin (3.52 ± 1.05 vs. 3.32 ± 2.13 × 10^9^/L) were significantly lower than normal controls (4.33 ± 1.52 × 10^9^/L) [34]. Therefore, evidence is lacking to support the efficacy of IVIG for total lymphocytes in patients with KD. Although we revealed lower segmented neutrophils after IVIG treatment in our research, it has been reported that IVIG may induce polymorphonuclear cells in the peripheral blood [35].

This study has certain limitations. First, since this is a study in methylation, whether the findings remain constant over mRNA levels requires further study. The transcription of the *FcγR2B* gene is not only regulated by promoter methylation but also by regulatory elements with silencer activity [36,37]. Second, *FcγR2B* methylation was not measured for patients without IVIG infusion because IVIG is the standard treatment for KD. Perhaps due to the presence of CAA, patients were more willing to participate in regular follow-up visits, and we could also collect blood samples, which may have caused a selection bias. We enrolled the cases with all KD1–5 samples younger than the expected average age of KD, reflecting selection bias in this cohort study. Nationwide population-based cohort studies have surveyed KD children with mean age of 1.57 years and 16.72 months in Taiwan [38,39]. The validation of *FcγR2B* methylation in one CpG site was also too limited to explain IVIG resistance or its long-term hypermethylation.

In summary, this study is the first to show that *FcγR2B* may serve as an indicator of IVIG resistance in KD1-3, as well as the first to report long-term epigenetic alterations in the *FcγR2B* methylation of KD.

## Figures and Tables

**Table 1:** (**A**) Basal characteristics of patients with Kawasaki disease and controls. (**B**) Characteristics of controls.

**A**			
	**Kawasaki Disease** ***n* = 76**	**Controls** ***n* = 40**	***p***
Age	1.79 ± 0.14	2.29 ± 0.26	0.10
Gender Female/male	34/42	19/21	0.78
Data are presented as mean with standard errors.			
**B**			
**Diagnosis**	**Number**	**Diagnosis**	**Number**
Low respiratory infection/bronchitis, bronchiolitis	6	Acute gastritis	1
Low respiratory infection/pneumonia, bronchopneumonia	10	Rotavirus infection	1
Upper respiratory infection/tonsillitis, pharyngitis	5	Bacteremia	1
Influenza A	1	Urinary tract infection	1
Acute otitis media	3	Acute enteritis with salmonella infection	1
Acute sinusitis	4	Hand-foot-mouth disease	1
Croup	1	Infectious mononucleosis	1
Acute mastoiditis	1	Herpetic gingivostomatitis	1
		Acute arthritis	1

## Data Availability

The data are available from the author upon request.
